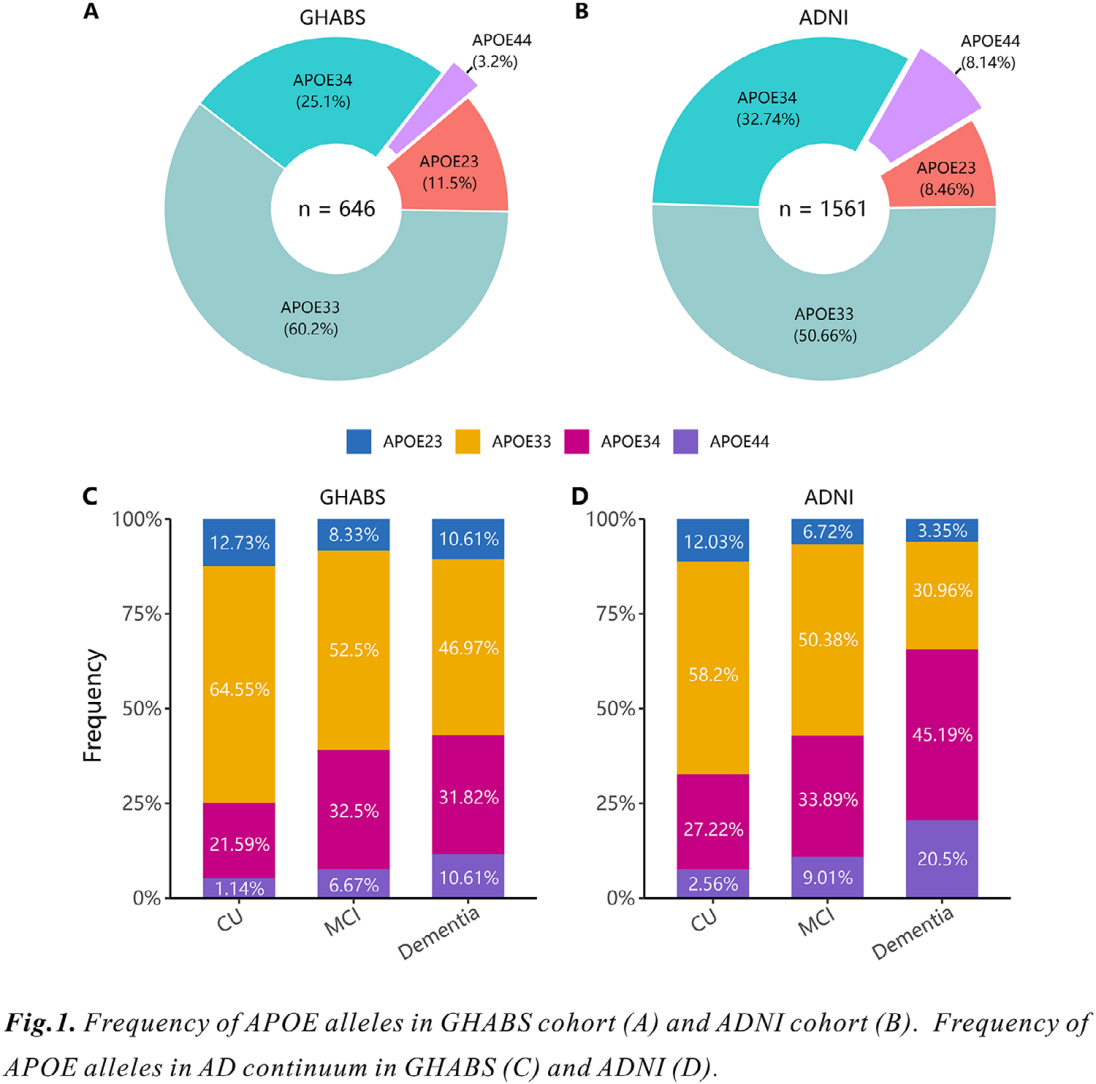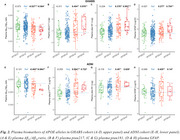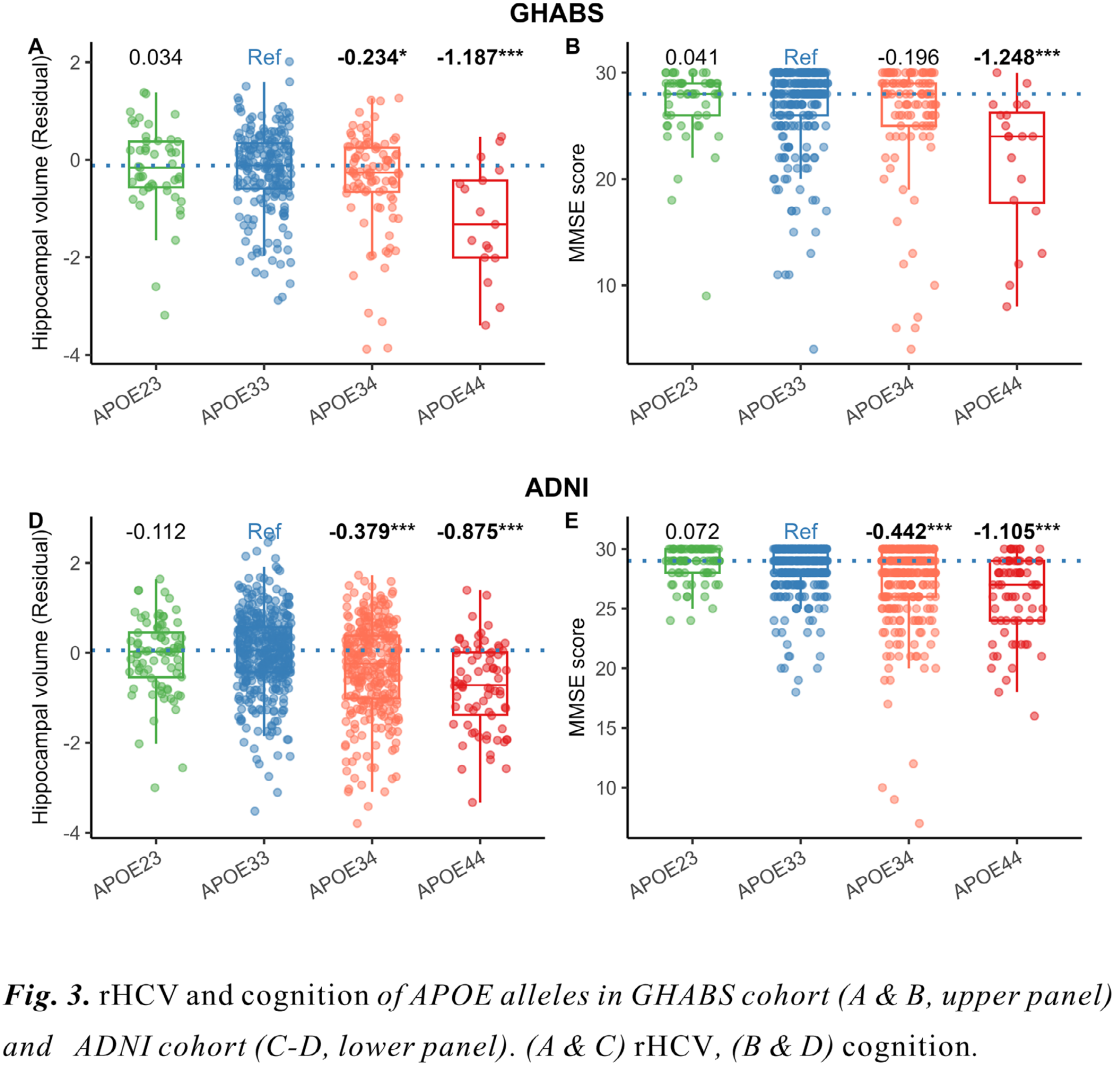# Comparisons of APOE‐ε4‐related Alzheimer's Disease Pathophysiology between Chinese and American Aging population

**DOI:** 10.1002/alz70856_101846

**Published:** 2025-12-25

**Authors:** Zhen Liu, Dai Shi, Anqi Li, Guoyu Lan, Laihong Zhang, Mingxing Jiang, Tengfei Guo

**Affiliations:** ^1^ Institute of Neurological and Psychiatric Disorders, Shenzhen Bay Laboratory, Shenzhen, Guangdong, China; ^2^ The Seventh Affiliated Hospital, Sun Yat‐sen University, Shenzhen, Guangdong, China; ^3^ Shenzhen Bay Laboratory, Shenzhen, Guangdong, China; ^4^ Institute of Biomedical Engineering, Shenzhen Bay Laboratory, Shenzhen, China; ^5^ Peking University Shenzhen Graduate School, Shenzhen, Guangdong, China

## Abstract

**Background:**

Apolipoprotein E‐ε4 (APOE‐ε4) is a major genetic risk factor for sporadic Alzheimer's disease (AD). Previous studies suggested that APOE‐ε4 homozygotes represent a genetic form of AD, and APOE4 appears to have a more pronounced effect on the East Asian aging population. However, it is still not fully understood whether APOE‐ε4 homozygosity shows more abnormal AD biomarker changes in East Asian older adults compared to the Caucasian aging population.

We compared APOE Genotype and AD risk in the Greater‐Bay‐Area Healthy Aging Brain Study (GHABS) cohort and the Alzheimer's Disease Neuroimaging Initiative (ADNI) cohort.

**Method:**

We identified 742 Chinese and 1561 American older adults from the GHABS and the ADNI cohorts, respectively. The frequency of APOE alleles was compared in the GHABS and ADNI cohorts. Furthermore, 440 Age‐ and sex‐matched GHABS participants and 870 ADNI participants were compared in plasma Aβ42/Aβ40 ratio, phosphorylated tau217 (ptau217), ptau181, and glial fibrillary acidic protein (GFAP), residual hippocampal volume (rHCV), and cognition, using the generalized linear models, controlling for age and sex. The Cohen's D effect size of each APOE genotype from the reference group APOE‐ε3ε3 was calculated.

**Result:**

Chinese older adults (GHABS) had lower percentages of APOE‐ε3ε4 (25.1% vs. 32.7%, Figure 1) and APOE‐ε4ε4 (3.2% vs. 8.14%, Figure 1) than American individuals (ADNI). Among different clinical groups, GHABS participants also showed less APOE‐ε3ε4 and APOE‐ε4ε4 compared to ADNI participants across CU, MCI, and dementia groups. In the comparison of the APOE‐ε3ε3 individuals, GHABS APOE‐ε4ε4 individuals showed more abnormal changes in plasma ptau217 (Cohen's d: 0.976 Vs. 0.722), plasma ptau181 (Cohen's d: 0.953 Vs. 0.629), plasma GFAP (Cohen's d: 0.754 Vs. 0.147), rHCV (Cohen's d: ‐1.187 Vs. ‐0.875), and cognition (Cohen's d: ‐1.248 Vs. ‐1.105) than ADNI APOE‐ε4ε4 participants.

**Conclusion:**

Less APOE‐ε4 carriers were observed in Chinese older adults than in American older adults. However, more detrimental effects of APOE‐ε4ε4 were found in plasma biomarkers, hippocampal atrophy, and cognitive decline among Chinese older adults than American older adults. These findings revealed the characteristics of the APOE‐ε4 allele and its influence on AD pathophysiology among the Chinese and American aging population.